# Flexible Deep-Brain Probe for High-Fidelity Multi-Scale Recording of Epileptic Network Dynamics

**DOI:** 10.3390/mi16060661

**Published:** 2025-05-30

**Authors:** Dujuan Zou, Lirui Yang, Guopei Zhou, Yan Zhang, Zhenyu Liang, Ziyi Zhu, Yanyan Nie, Huiran Yang, Zhitao Zhou, Liuyang Sun, Xiaoling Wei

**Affiliations:** 1 State Key Laboratory of Transducer Technology, Shanghai Institute of Microsystem and Information Technology, Chinese Academy of Sciences, Shanghai 200050, China; dujuan@mail.sim.ac.cn (D.Z.); yanglirui24@mails.ucas.ac.cn (L.Y.); boblezhou@gmail.com (G.Z.); zhangyan1@mail.sim.ac.cn (Y.Z.); lzy0221@mail.sim.ac.cn (Z.L.); zhuzy22@mail.sim.ac.cn (Z.Z.); hryang@mail.sim.ac.cn (H.Y.); ztzhou@mail.sim.ac.cn (Z.Z.); 2 School of Graduate Study, University of Chinese Academy of Sciences, Beijing 100049, China; liuyang.sun@mail.sim.ac.cn; 3 Wuhan Research Institute of Posts and Telecommunications, Wuhan 430074, China; 4 Shanghai Laboratory Animal Research Center, Shanghai 201203, China; nieyanyan@slarc.org.cn; 5 2020 X-Lab, Shanghai Institute of Microsystem and Information Technology, Chinese Academy of Sciences, Shanghai 200050, China

**Keywords:** flexible probe, wideband neural recording, AP-HFO coupling, seizure mechanisms

## Abstract

Epilepsy is a complex neurological disorder characterized by abnormal neural synchronization and interactions between local foci and global brain networks during seizures. Understanding seizure mechanisms across multiple scales is essential for advancing our understanding of epileptic network dynamics and guiding personalized treatment strategies. However, neural recording technologies are limited by insufficient spatial resolution, signal fidelity, and the inability to simultaneously capture network- and cellular-level dynamics. To address these limitations, we developed a high-density, flexible deep-brain probe with excellent mechanical compliance and wideband recording capabilities, enabling high-fidelity recordings of high-frequency oscillations (HFOs, 80–500 Hz) and action potentials (APs). Using a pentylenetetrazol (PTZ)-induced epilepsy model, we identified distinct spatiotemporal dynamics of HFOs and APs across epileptic stages, indicating that CA3 plays a key role in seizure onset, while CA1 is crucial for propagation. AP-HFO coupling analysis further uncovered neuronal heterogeneity, offering insights into the diverse roles of neurons in epileptic networks. This study highlights the potential of a flexible deep-brain probe for advancing epilepsy research and guiding personalized therapeutic interventions.

## 1. Introduction

Seizures involve abnormal synchronization of neuronal activity and dynamic interactions between local foci and global brain networks [1,2]. These processes, which underlie epileptic seizure initiation and propagation, reflect pathological changes at both network and cellular levels [3]. Investigating these mechanisms is essential for understanding the pathological dynamics of epileptic seizures, which may provide valuable insights for enhancing the theoretical foundation of seizure focus localization and developing personalized therapeutic strategies [4,5] However, progress in this area has been hindered by the technical limitations of neural recording technologies [6,7].

Tools such as scalp electroencephalography (EEG), electrocorticography (ECoG), and stereotactic electroencephalography (SEEG) have made significant contributions to epileptic focus localization [8]. However, each approach has inherent limitations. EEG, as a non-invasive technique, offers a global view of brain activity but suffers from low spatial resolution and limited access to deep brain structures [9,10,11]. ECoG improves signal resolution but is confined to the cortical surface, requires a large craniotomy, and leaves deeper regions inaccessible [12,13]. SEEG, while enabling recordings from deep brain regions, utilizes relatively large electrodes (typically 0.8–1.3 mm in diameter) with limited spatial resolution and channel density [8,14,15]. To address these limitations, we summarized the key metrics of commercial epilepsy electrodes in Table S1.

HFOs have emerged as critical biomarkers of epileptic foci, reflecting abnormal synchronization of neuronal populations and playing a significant role in seizure initiation and propagation [16]. At the cellular level, action potentials (APs) provide direct insights into neuronal spiking activity, revealing the fundamental mechanisms driving epileptic seizures [17,18]. The comprehensive study of both HFOs (80–500 Hz) and APs (typically > 300 Hz) requires high temporal resolution and close proximity to neural tissue at a scale that conventional clinical electrodes, including SEEG, cannot consistently provide [19,20,21].

Microscale implantable neural probes, with significantly smaller dimensions and higher electrode density than clinical SEEG, have addressed these recording challenges by enabling intimate interface with neural tissue at the cellular level. These probes successfully capture both HFOs and APs signals, providing complementary perspectives on network-level and cellular-level activity [22]. However, conventional rigid probes present significant limitations for long-term monitoring, including enhanced tissue damage due to mechanical mismatch, inflammatory responses, and signal degradation over time [23].

Flexible deep-brain probes provide a promising solution to these challenges. Utilizing substrates such as polyimide (PI), parylene, and SU-8, which have Young’s moduli ranging between 2 and 10 GPa, flexible probes achieve low bending stiffness and offer excellent tissue compliance. This design minimizes tissue damage and strain caused by brain motion, ensuring stable, long-term recordings [24,25]. These mechanical advantages, coupled with preserved wideband recording capabilities, make flexible probes ideal for sustained multi-scale investigations into seizure mechanisms over extended periods.

Bonaccini Calia et al. developed flexible graphene depth neural probes (gDNP, 10 μm thick), validating the feasibility of flexible electrodes for recording multi-band neural signals. Their research successfully achieved full-bandwidth recording from DC shifts to high-frequency oscillations, and demonstrated the spatiotemporal relationship between DC signals and seizures, offering new perspectives for understanding seizure mechanisms [26]. Their research primarily focused on the relationship between low-frequency signals and seizures, establishing a technical foundation for exploring the role of different frequency bands in epilepsy.

Yu Wang et al. utilized 8 μm thick flexible deep-brain electrodes, combining recording and stimulation functions to achieve long-term stable neuronal discharge recordings. By analyzing changes in the spatial encoding capacity of place cells before and after epilepsy, their research revealed the mechanisms by which epilepsy affects hippocampal cognitive function [27]. This work provided a unique perspective for understanding epilepsy from the cellular function level, focusing on changes in cellular-level neural activity.

Our previous research has also explored the application of flexible deep-brain electrodes in epilepsy monitoring. Cheng Q et al. used ultra-thin flexible probes (2 μm thick) to successfully extract gamma band (30–100 Hz) signals, verifying synchronization between CA1 and CA3 brain regions during epileptic discharges, and demonstrating changes between interictal and ictal periods through the analysis of neuronal discharge full width at half maximum (FWHM) features [28]. This study primarily focused on the relationship between gamma rhythms and seizures, as well as changes in neuronal discharge characteristics, providing important evidence of network synchronization for epilepsy mechanism research.

In this study, we developed a high-density PI based flexible probe optimized for high-fidelity recordings in deep brain regions. Using a PTZ-induced epilepsy model, we explored the spatiotemporal dynamics of HFOs and APs, revealing their distinct roles across epileptic stages, as well as the coupling between HFOs and APs, which reflects distinct mechanisms of neuronal synchronization and seizure propagation. This work not only provides new insights into epilepsy mechanisms but also establishes a methodological foundation for multi-scale epilepsy research.

## 2. Materials and Methods

### 2.1. Electrode Surface Modification

The dual-sided flexible probes were fabricated using MEMS-compatible micro- and nanomanufacturing techniques. A 4-inch silicon wafer (n-type 0.005 V·cm, XiaMen LuYuan Science and Technology, Xiamen, China) was used as the substrate, with a 200 nm silicon dioxide insulating layer formed via wet oxidation. The fabrication process involved precise photolithography, multilayer metal deposition, and dry etching to achieve high-density interconnects and dual-sided conductive pathways. For the photolithography steps, positive photoresist (LC100A, Rohm & Haas Electronic Materials (Shanghai) Co., Ltd., Shanghai, China) was spin-coated at 3000 rpm for 45 s, followed by soft baking at 110 °C for 60 s. The exposure power was 13.5 mJ/cm^2^, and development was carried out in 2.38% TMAH (NMD-3, Tetramethylammonium hydroxide [75-59-2], Jiangyin Jianghua Micro-Electronic Materials Co., Ltd., Jiangyin, China) solution for 90 s. Key steps included the patterning of Cr/Ni (5 nm/100 nm) sacrificial layers via electron beam evaporation, deposition of Cr/Au (10 nm/150 nm) for the bottom electrodes via electron beam evaporation, and application of polyimide (PI-2610, HD Microsystems, Parlin, NJ, USA) as the bottom insulating layer, which was spin-coated at 3000 rpm for 60 s and cured at 350 °C using a hard aluminum mask. The polyimide layer was patterned using reactive ion etching (RIE) as the dry etching process. The metal via layer consisted of Cr/Au (10 nm/350 nm) deposited via electron beam evaporation, while the metal routing layer was formed with Cr/Au/Cr (10 nm/150 nm/10 nm) also via electron beam evaporation. A Cr/Ni/Au (5 nm/100 nm/50 nm) layer was used for solder pads to ensure stable connections during packaging. The top insulating layer and top electrode layer (Cr/Au, 10 nm/150 nm) were prepared using similar processes to those of their bottom counterparts. The detailed fabrication process of the probes is illustrated in Figure S3. In the final release step, we applied a commercial nickel etchant (Transene Nickel Etchant Type TFB, Transene Company Inc., Danvers, MA, USA) to the flexible electrode tip for 15 min to selectively etch the Ti/Ni sacrificial layer. After etching, the sample was thoroughly rinsed with deionized (DI) water and subjected to ultrasonic cleaning to ensure complete removal of residual etchant. Once the sacrificial layer was completely removed, the four shanks at the flexible electrode tip were released from the silicon substrate. We then carefully moved these flexible shanks to the side and performed dicing on the remaining silicon wafer to complete the release process and separate the individual probes. To enhance their electrochemical performance, the electrode surfaces were electroplated with a conductive polymer using an electrodeposition process. The electroplating solution was prepared by mixing 60 μL of EDOT (3,4-ethylenedioxythiophene, Sigma-Aldrich, St. Louis, MO, USA, product number 483028), 2 mL of PSS (poly(sodium 4-styrenesulfonate), Sigma-Aldrich, St. Louis, MO, USA, product number 243051), and 1 mL of deionized (DI) water. The solution was thoroughly mixed using a vortex mixer to ensure homogeneity.

Electrodeposition was performed using a PSTrace4 electroplating system (version 5.11, PalmSens BV, Houten, The Netherlands) in a three-electrode configuration. The reference electrode was Ag/AgCl, the counter electrode was a platinum wire, and the working electrode was the channel to be plated, with a ground electrode connected to grounding equipment for electrical stability. The process was conducted in cyclic voltammetry (CV) mode with a voltage range of 0.05 V to 0.95 V and a scan rate of 50 mV/s, applied for 30 cycles. These parameters were selected to optimize the conductivity and stability of the PEDOT:PSS electroplating, ensuring sufficient thickness and enhanced electrochemical performance.

### 2.2. Implantation of Flexible Neural Probes

All animal procedures were carried out at Shanghai Laboratory Animal Research Center, Shanghai, China. All animal-related experiments were approved by the Institutional Animal Care and Use Committee of Shanghai Lab. Animal Research Center (approval number: PA202301002). Male C57BL/6JGpt mice (6–8 weeks old, Shanghai Yaokang Biotechnology Co., Ltd., Shanghai, China) were anesthetized with isoflurane (3% for induction, maintained at 1–1.5%) delivered in medical-grade oxygen and fixed in a rodent stereotaxic frame (RWD Life Science Co., Ltd., Shenzhen, China) with ear bars to immobilize the head. After shaving and sterilizing the scalp, the skull was exposed by incision and cleaned to ensure proper adhesion for dental cement fixation. Using a stereotaxic manipulator, a stainless steel wire (diameter: 100 μm) was implanted as a reference electrode at coordinates AP: −2.0 mm, ML: −2.0 mm, and DV: −2.0 mm relative to bregma. A flexible neural probe assembly, with four shanks aligned and temporarily bonded to a tungsten wire (diameter: 50 μm) using polyethylene glycol (PEG) (see Figure S10 for probe-wire assembly), was implanted at ML: 2.75 mm, AP: −2.45 mm, and DV: −3.0 mm from bregma. After insertion, the PEG was dissolved with sterile physiological saline, and the tungsten wire was removed. The probe was secured to the skull using dental cement (Super Bond C&B, Sun Medical Co., Ltd., Moriyama, Kyoto, Japan), completing the implantation process [29].

### 2.3. Epileptic Mouse Model Induction

Epilepsy was induced in male C57BL/6JGpt mice that had undergone probe implantation by intraperitoneal injections of PTZ (37.5 mg/kg, dissolved in physiological saline; Sigma-Aldrich, P6500, USA) every two days for a total of 14 injections. During the induction period, body weight was monitored regularly to ensure all mice remained within a healthy weight range. After each injection, behavioral responses were recorded for 30 min using a video monitoring system, and seizure severity was evaluated using the Racine scale [30]. The onset time and progression of seizures were documented for each mouse. This protocol successfully established an epileptic mouse model for subsequent electrophysiological recordings in hippocampal regions (CA1 and CA3).

In addition, we established another mouse model of epilepsy using stereotaxic injection of KA (kainic acid, Sigma-Aldrich, K0250, USA) into the brain. Mice were injected with 1 μL of KA solution at a concentration of 1 μg/μL. Neural signals were recorded after the injection while monitoring the seizure activity of the mice. Data from the KA-induced mouse model are presented in Figure S5, whereas the main text primarily focuses on data from the PTZ-induced mouse model.

### 2.4. Neural Signal Recording

Neural activity was recorded using flexible neural probes connected to a 128-channel headstage (Intan Technologies, Los Angeles, CA, USA). Before each PTZ injection, neural signals were recorded for 10 min to monitor baseline activity. Following the intraperitoneal injection of PTZ, neural recording continued without interruption, and the time at which the mouse was returned to the observation cage was noted. Simultaneously, video recording was initiated to synchronize behavioral observations with neural signal data. Neural activity was recorded for an additional 30 min post-injection, during which the progression of seizures and behavioral changes was monitored. By the end of this period, most mice exhibited no further epileptic behaviors and began returning to normal activity patterns.

### 2.5. HFOs and AP Analysis in Neural Signals

The recorded neural signals were processed using Offline Sorter (Plexon Inc., Offline Sorter v4, Dallas, TX, USA) to classify APs. The raw signals were first high-pass filtered at 250 Hz using a Butterworth filter to isolate spike waveforms. Threshold-based detection was then applied to preliminarily identify spikes. To further classify APs, principal component analysis (PCA) was performed on the detected spike waveforms, and clustering methods were used to differentiate units. The quality of unit separation was evaluated using inter-spike interval (ISI) histograms and autocorrelograms (ACG), enabling the identification of single-unit activity (SUA) or multi-unit activity (MUA). The spike data and corresponding waveforms were then exported for subsequent analysis. HFOs were identified using custom Python scripts. The raw neural signals were first bandpass filtered within the 80–500 Hz range to isolate high-frequency components. Adaptive filtering was then applied to remove motion artifacts and environmental noise. HFOs were defined as continuous oscillations lasting 4–12 cycles, with amplitudes exceeding two standard deviations (SD) of the baseline signal. HFO events recorded during baseline and ictal periods from the same recording channel were pooled into a single dataset for further classification and analysis.

The pooled dataset was analyzed using Python-based algorithms. Dimensionality reduction and visualization were performed using the uniform manifold approximation and projection (UMAP) algorithm, while clustering was conducted using the Leiden algorithm to group HFOs based on high-dimensional features. To further refine the classification, a gaussian mixture model (GMM) was applied to the clustered data, enabling the differentiation of physiological and pathological HFOs through quantitative analysis and visualization. After identifying pathological HFOs, timestamps of APs and HFOs were synchronized. A custom Python program was then used to calculate firing rate correlations and assess the statistical significance of the relationship between APs and pathological HFOs. All signal processing and statistical analyses were conducted using Python (version 3.8).

## 3. Results

### 3.1. High-Resolution Recording Capability of Flexible Probes

**Figure 1:**
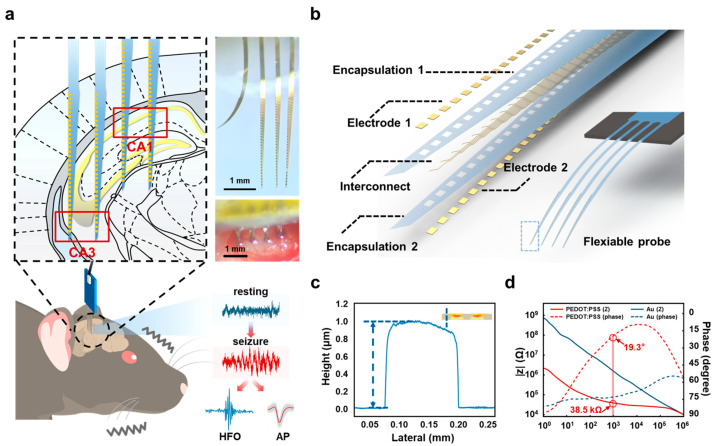
Design, implantation, and characterization of the high-density flexible probe for hippocampal recordings in an epilepsy model. (**a**) Design and implantation of the flexible probe for hippocampal recordings. Representative neural signals recorded during resting (blue) and seizure (red) states are shown, with seizure signals further decomposed into high-frequency oscillations (HFOs) (blue) and action potentials (APs) (red), demonstrating the probe’s ability to capture epilepsy-related signals. (**b**) Structural design of the dual-sided flexible probe. (**c**) Thickness measurement of the flexible probe. The total thickness of the dual-sided probe was measured using a profilometer, revealing an ultrathin structure with a thickness of only 1 μm. (**d**) Impedance characterization of the electrode before and after electrochemical deposition. The initial gold electrode surface was modified by electroplating with PEDOT:PSS. After electroplating, the impedance magnitude (|Z|) was significantly reduced to 38.5 kΩ, while the phase angle decreased to 19.3°.

To enable high-resolution neural recordings in the hippocampus, we developed a four-shank ultrathin, dual-sided flexible probe, as shown in Figure 1. This probe was specifically designed to overcome the limitations of traditional rigid probe designs by providing enhanced flexibility and multi-channel recording capabilities across interconnected brain regions. The flexible probes were fabricated using a PI substrate with a thickness of 1 μm, known for its excellent mechanical compliance and flexibility. Its bending stiffness was calculated as 2.36 × 10^−14^ N·m^2^ (Figure S3), making it highly suitable for neural interface applications. The probe includes 128 recording sites distributed across four shanks, with 32 sites per shank. The vertical spacing between recording sites is 75 μm, while the shank-to-shank spacing is 500 μm (Figure 1a), enabling simultaneous recordings across the CA1 and CA3 regions of the hippocampus.

The dual-sided design increases the contact area with neural tissue, improving signal quality, enhancing fabrication yield (Figure S11), and enabling the detection of both HFOs and APs from multiple channels [31]. Moreover, the probe’s flexibility minimizes mechanical stress and tissue damage during implantation, ensuring stable, high-fidelity recordings from deep brain regions. To further validate the minimal tissue damage caused by the flexible probe, we conducted immunohistochemical staining of brain tissue at the implantation site. Compared to rigid probes, the flexible probe showed significantly reduced activation of glial cells, as indicated by lower GFAP and Iba1 fluorescence intensities. Detailed results are provided in Figure S6. These features allow for simultaneous multi-site recordings of HFOs and APs, providing unique insights into the spatiotemporal organization of epileptic activity across hippocampal subregions.

In summary, the flexible probe’s high-density configuration, multi-regional coverage, and ability to detect both HFOs and APs at high resolution establish it as a powerful tool for studying hippocampal network dynamics during baseline and epileptic states. Importantly, the probe’s high-resolution capability captures subtle changes in neural activity, enabling detailed spectral analyses to investigate frequency-specific characteristics of epileptic activity. These unique capabilities form the foundation for the subsequent spectral and dynamic analyses presented in this study.

### 3.2. Spectral Analysis of Neural Activity Across Epileptic Stages

**Figure 2:**
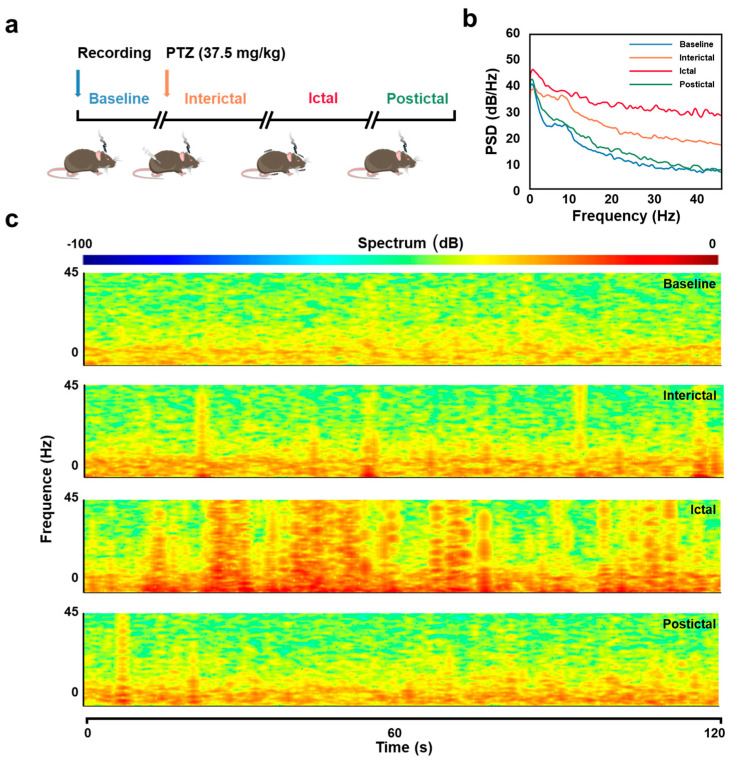
Neural activity analysis during baseline, interictal, ictal, and postictal periods in a pentylenetetrazol (PTZ)-induced epilepsy model. (**a**) Experimental procedure for PTZ-induced epilepsy model. Mice were implanted with flexible probes for hippocampal recordings. Baseline signals were recorded before the intraperitoneal injection of PTZ (37.5 mg/kg). (**b**) Power spectral density (PSD) analysis for each of the four periods. (**c**) Time–frequency spectrograms of neural signals. Spectrograms (0–120 s, 0–45 Hz) for each of the four periods.

Epileptic seizures are characterized by abnormal neural synchronization and network excitability, which are reflected in distinct spectral patterns across different frequency bands. To investigate these changes, hippocampal neural signals recorded during four stages of PTZ-induced epilepsy (baseline, interictal, ictal, and postictal) were analyzed for their spectral characteristics (Figure 2).

The power spectral density (PSD) analysis revealed significant changes in the 0–45 Hz range across these stages (Figure 2b). During the ictal phase, PSD exhibited a marked increase in low-frequency power (<30 Hz) compared to baseline and postictal phases, reflecting heightened network synchronization during seizures. The interictal phase displayed intermediate power levels, showing a gradual transition between baseline and ictal states. Time–frequency spectrograms further illustrated these dynamics, with the ictal phase showing sustained increases in low-frequency power, while baseline and postictal phases maintained weaker, stable power distributions (Figure 2c). In contrast, interictal periods demonstrated transient bursts of low-frequency power, indicating intermediate network activity.

While these low-frequency spectral changes provide a broad overview of epileptic network dynamics, they lack the specificity required to identify pathological biomarkers essential for understanding seizure mechanisms. HFOs are increasingly recognized as reliable indicators of pathological synchronization, offering finer-scale insights into seizure activity [32]. To bridge this gap, we conducted HFO detection and clustering analysis to investigate their characteristics during baseline and epileptic stages, providing a more detailed perspective on the neural dynamics underlying epilepsy.

### 3.3. Detection, Clustering, and Characterization of HFOs Across Baseline and Epileptic States

**Figure 3:**
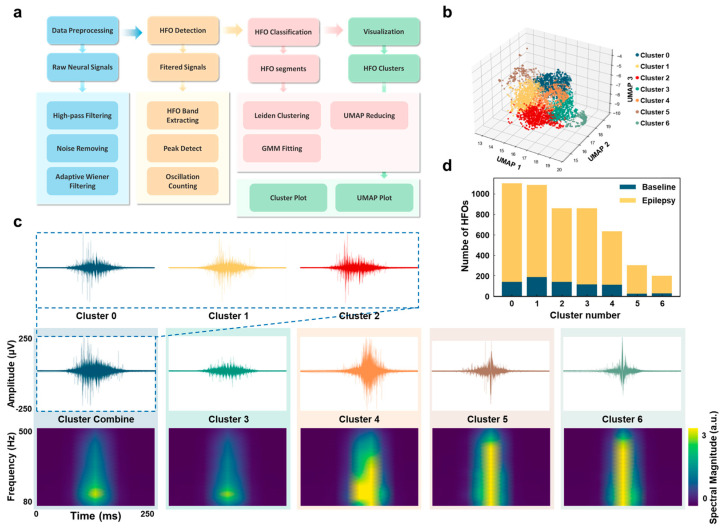
Characterization and clustering of HFOs in baseline and epileptic states. (**a**) Workflow for HFO detection and clustering. The diagram illustrates the steps for processing raw neural signals, including filtering, detection, clustering, and visualization of HFOs. (**b**) UMAP-based clustering of HFOs. HFOs detected before PTZ injection (baseline, 3 min) and during epileptic seizures (3 min) were pooled and classified into seven clusters (Clusters 0–6) using UMAP dimensionality reduction and clustering. (**c**) Waveform and time-frequency characteristics of HFO clusters. Superimposed waveforms of Clusters 0, 1, and 2 are shown in the first panel. The second panel presents the combined waveforms of Clusters 0, 1, and 2 (“Cluster Combined”) alongside individual waveforms of Clusters 3 to 6. The third panel shows the time–frequency spectrograms corresponding to the waveforms in the second panel, with each HFO segment representing a 250 ms window. (**d**) Distribution of HFO clusters across baseline and epileptic states. Bar plots showing the relative occurrence of HFOs in each cluster (Clusters 0–6) during baseline and epileptic states, highlighting differences in HFO distribution between the two conditions.

HFOs were detected from hippocampal neural signals recorded 3 min before PTZ injection (baseline) and during the ictal phase following PTZ injection. Using an automated detection and clustering workflow (Figure 3a), HFO waveforms were pooled and clustered into seven distinct groups (Clusters 0–6, Figure 3b). Waveform and spectral analyses revealed that Clusters 0, 1, and 2 exhibited highly similar spectral energy distributions and waveforms. Due to their strong similarities, these clusters were manually combined into a single group (“Cluster Combined”) for subsequent analysis. In contrast, Clusters 5 and 6 were excluded from further analysis due to their atypical spike-like waveforms that displayed irregular peak distributions rather than rhythmic oscillations, abnormal amplitude changes, and flat spectral profiles in the HFO frequency range (80–500 Hz). These characteristics, combined with their significantly lower occurrence frequency, strongly suggested they represented detection artifacts rather than genuine neural oscillations [33].

Quantitative analyses showed a substantial increase in the total number of HFOs during the ictal phase compared to the baseline period (Figure 3d), with all remaining clusters (Cluster Combined, Cluster 3, and Cluster 4) contributing to this increase. Cluster Combined and Cluster 3 displayed typical HFO waveforms and spectral characteristics within the 80–200 Hz range, reflecting both physiological synchronization related to normal network activity and pathological synchronization associated with epileptic seizures. In contrast, Cluster 4 exhibited distinct features, including higher amplitudes and spectral energy concentrated above 200 Hz, consistent with pathological HFOs linked to epileptic activity and heightened neuronal synchronization [34,35].

A small proportion of Cluster 4 HFOs was also detected during the baseline period, potentially reflecting ongoing pathological activity in the chronic epileptic state of the experimental mice. This suggests that while Cluster Combined and Cluster 3 are more broadly associated with network synchronization mechanisms, Cluster 4 requires more stringent conditions for its generation, such as higher levels of neuronal coupling and specific network configurations.

To gain deeper insights into the mechanisms underlying HFO generation and their coupling relationships, we extended our analyses to explore the temporal dynamics of HFOs during seizure progression. This also allowed us to investigate their interplay with action potentials (APs) as a complementary measure of neuronal activity, adding another layer of understanding to HFO dynamics.

### 3.4. Dynamic Characteristics of HFOs and APs Across Epileptic Stages

**Figure 4:**
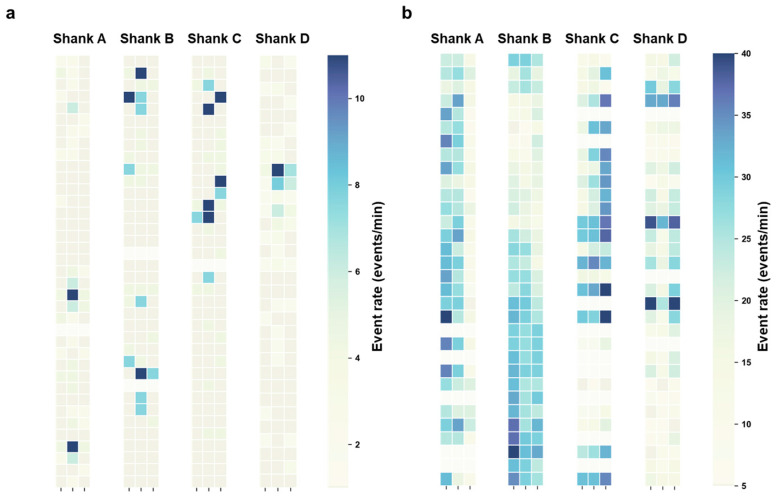
Heatmap comparison of 3 min HFO firing rates. (**a**) Normal mice without epilepsy induction. (**b**) Mice with generalized epilepsy induced by intraperitoneal injection of PTZ. Each pixel in the heatmap represents the HFO firing rate for a single channel over a 1 min period. The 128 channels are distributed across four shanks (A, B, C, and D), with 32 channels per shank.

To investigate the dynamic characteristics of HFOs and APs during epileptic stages, we compared the firing rates of HFOs in normal and PTZ-induced epileptic mice within a 3 min time window (Figure 4). This analysis focused on the entire hippocampal region, spanning 128 channels distributed across four shanks, to highlight spatial differences in network activity (Figure S13). The heatmap comparison revealed that in normal mice (Figure 4a), HFO events were sporadic, with firing rates typically in the single digits per minute and without consistent spatial patterns. By contrast, in PTZ-induced epileptic mice (Figure 4b), the overall HFO firing rate was significantly elevated, with localized regions in CA3 and CA1 showing particularly high firing rates, reaching 30 HFO events per minute on certain channels. These findings indicate enhanced network synchronization and hyperexcitability in specific hippocampal regions during epilepsy.

**Figure 5:**
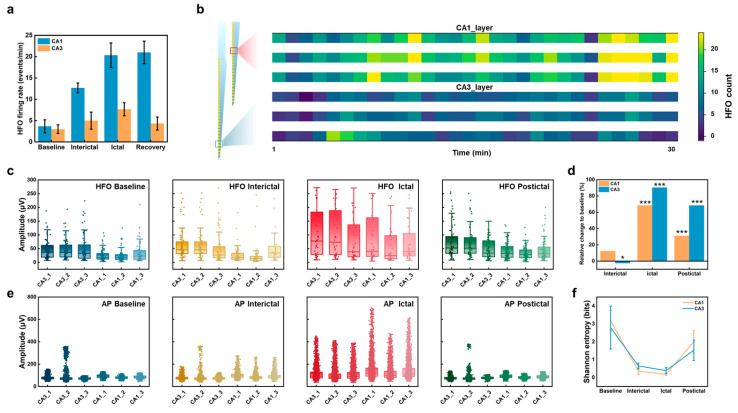
Dynamic changes in HFO and AP activity across epileptic stages in CA1 and CA3 regions. Data from baseline, interictal, ictal, and postictal phases were extracted as 2 min segments from a continuous 30 min recording following PTZ injection. (**a**) HFO firing rates across epileptic stages. Bar plots showing HFO firing rates during baseline, interictal, ictal, and recovery phases. (**b**) Heatmaps of HFO firing rates post-PTZ injection. Heatmaps displaying HFO firing rates from the continuous 30 min recording divided into 30 1 min intervals, showing three channels in the CA1 region (CA1_1, CA1_2, and CA1_3) and three in the CA3 region (CA3_1, CA3_2, and CA3_3). (**c**,**e**) HFO and AP amplitudes across epileptic stages. (**d**) Percentage change in HFO amplitudes relative to baseline. CA1 and CA3 regions show distinct percentage increases during ictal and postictal phases, with CA1 exhibiting higher relative changes during propagation. Statistical significance was assessed using the Mann–Whitney U test due to the non-parametric nature of the data. Asterisks indicate significance levels: * *p* < 0.05, *** *p* < 0.001. (**f**) Shannon entropy of APs across epileptic stages. Boxplots display the Shannon entropy for AP amplitudes in CA1 and CA3 regions, revealing stage-specific decreases during ictal phases and partial recovery postictally.

We further analyzed the spatiotemporal dynamics of HFO firing rates during the entire 30 min continuous recording of the epileptic process (Figure 5a,b). To investigate layer-specific differences, we selected three representative channels from the CA3 and CA1 regions. The results revealed distinct temporal patterns: In CA3, HFO firing rates progressively increased from baseline to the ictal phase, peaking early (10–15 min post-PTZ injection) and partially decreasing during the postictal phase, suggesting CA3′s early involvement in initiating epileptic activity. In contrast, CA1 firing rates showed a delayed but more pronounced increase during the later ictal phase (25–30 min), reflecting its role in seizure propagation. The heatmaps (Figure 5b) further illustrate these spatiotemporal dynamics, highlighting CA3 as a potential initiator and CA1 as a key contributor during seizure progression. For analytical purposes, this continuous 30 min recording was divided into 30 1 min intervals to better visualize the dynamic changes across different phases.

These spatiotemporal differences in HFO firing rates suggest that distinct hippocampal regions and layers contribute differently to the initiation and propagation of epileptic activity. However, firing rates alone cannot fully capture the extent of network excitability and pathological synchronization during seizures. To gain deeper insights into these processes, we next analyzed the amplitudes of both HFOs and APs across different epileptic stages by extracting 2 min segments from each of the baseline, interictal, ictal, and postictal periods within the continuous recording.

HFO and AP amplitudes exhibited stage-dependent patterns (Figure 5c–e). During the ictal phase, HFO amplitudes in both CA1 and CA3 regions significantly increased compared to baseline (*p* < 0.01). Specifically, CA1 amplitudes increased by 90.27%, from 35.6 μV at baseline to 67.7 μV during the ictal phase, while CA3 amplitudes increased by 68.38%, from 30.2 μV to 50.9 μV. These results reflect heightened excitability and pathological synchronization of the neuronal network during seizures. In the postictal phase, HFO amplitudes partially decreased (CA1: 49.3 μV; CA3: 40.1 μV) but remained significantly higher than baseline (*p* < 0.05), suggesting residual pathological activity in the network.

The Shannon entropy analysis of AP amplitudes (Figure 5f) further supported the findings from HFO analysis. During the baseline period, Shannon entropy values were high in both CA1 and CA3 regions (CA1: 3.18 bits; CA3: 2.79 bits), reflecting high neuronal firing complexity. However, during the ictal phase, entropy significantly decreased (CA1: 0.18 bits; CA3: 0.39 bits), indicating reduced neuronal firing complexity and increased synchronization. In the postictal phase, entropy values partially recovered (CA1: 1.89 bits; CA3: 1.65 bits) but did not return to baseline levels, indicating persistent network abnormalities. These data suggest that the dynamic changes in HFO and AP reveal the profound impact of seizures on hippocampal network dynamics and highlight distinct activity patterns across different epileptic stages.

Through the analysis of HFO and AP amplitudes, we further revealed the changes in the neural network during epileptic activity. Our flexible probe approach builds upon previous significant contributions in the field of epilepsy research. Prior studies utilizing flexible neural interfaces have successfully demonstrated the capability to record multiple frequency bands of neural activity during epileptic events and provided valuable insights into seizure mechanisms through various analytical approaches. These works established important correlations between specific neural activities and epileptic phenomena [26,27]. Building on these foundations, our present work extends the analytical capabilities by simultaneously characterizing both HFOs and APs across the complete epileptic cycle. This comprehensive parametric analysis throughout baseline, preictal, ictal, and postictal phases provides detailed insights into how different neural signal components evolve and interact during epileptogenesis, seizure propagation, and termination. Our results not only confirm the findings from previous studies regarding altered neural dynamics during seizures but also reveal new layer-specific and temporal-specific patterns in hippocampal activity that contribute to our understanding of epileptic network mechanisms. To gain a deeper understanding of the relationship between HFOs and APs, we next explored the coupling dynamics between these two across different epileptic stages.

### 3.5. Coupling and Heterogeneity of HFOs and APs

**Figure 6:**
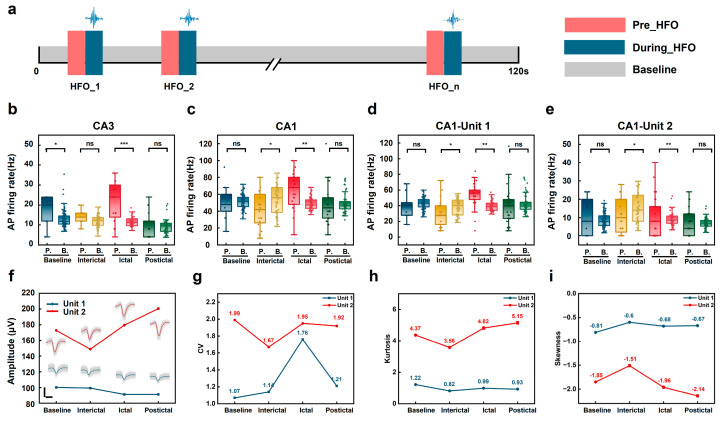
Differential AP-HFO coupling across epileptic stages and neuronal heterogeneity within a single recording channel. (**a**) Schematic representation of the time windows used for AP-HFO analysis. Each epileptic stage includes a 120 s representative recording period used to quantify AP-HFO coupling. Three time windows were defined relative to the HFO onset: pre-HFO (red, 250 ms before HFO onset), during HFO (blue, 250 ms), and the baseline (gray, stage-specific baseline). The baseline firing rates were calculated using a sliding window (window size: 5 s, step: 2 s) across the 120 s recording period in each epileptic stage, reflecting local firing rate dynamics for that phase. (**b**–**e**) Boxplots comparing pre-HFO AP firing rates with baseline firing rates across epileptic stages. (**d**,**e**) Boxplots showing data from two different units (Unit 1 and Unit 2) isolated from the same recording channel in CA1.The horizontal axis in these boxplots represents the following: P. represents pre-HFO AP firing rate, and B. represents baseline AP firing rate. Statistical analyses reveal differences in firing rates between pre-HFO and baseline periods, with changes becoming more pronounced as epileptic severity increases. Statistical comparisons between pre-HFO (250 ms windows) and baseline periods (calculated using 5 min sliding windows with 2 min steps) were performed using independent samples *t*-test, considered appropriate due to the limited temporal overlap and the substantial difference in window sizes. Significance levels: * *p* < 0.05, ** *p* < 0.01, *** *p* < 0.001, ns: not significant; effect sizes reported as Cohen’s d. (**f**) Representative waveforms for Unit 1 (blue) and Unit 2 (red), isolated from the same channel in CA1, shown across four epileptic stages (baseline, interictal, ictal, and postictal) with consistent time (100 µs) and z-amplitude scale (100 µV). Waveforms are displayed at their corresponding data points on the amplitude trend lines, illustrating the morphological changes through different epileptic stages. (**g**–**i**) Quantitative analysis of individual waveform characteristics across epileptic stages for both units: coefficient of variation (CV) (**g**), kurtosis (**h**), and skewness (**i**). Note the distinct patterns between units: Unit 1 exhibits maximum CV during ictal phase (1.76) with modest changes in distribution parameters, while Unit 2 shows more dynamic regulation with decreased CV during interictal periods (1.67) and pronounced increases in kurtosis during ictal (4.82) and postictal (5.15) phases, reflecting greater sensitivity to epileptic state transitions.

To further investigate the relationship between HFOs and APs, we analyzed AP firing rates during the pre-HFO period (250 ms before HFO onset) across different epileptic stages (Figure 6a). The analysis revealed stage- and layer-specific differences in AP-HFO coupling, as well as notable variability in coupling strength among neurons within the same recording location.

AP-HFO coupling was strongest during the ictal phase, with CA3 showing a significantly stronger association (*p* < 0.001, d = 1.974) compared to CA1 (*p* = 0.006, d = 0.627). This result is consistent with the established role of CA3 in early seizure propagation. During the interictal phase, coupling trends were weaker but still detectable, with CA1 showing a potential inhibitory effect on AP activity (*p* = 0.027, d = −0.586). By the postictal phase, no significant coupling was detected in either layer, indicating a return to baseline-like neuronal activity (Figure 6b,c).

At the neuronal unit level, waveform clustering identified two neuronal units (Unit 1 and Unit 2) from the same CA1 recording channel, each exhibiting distinct AP-HFO coupling characteristics (Figure 6d,e). During the ictal phase, AP-HFO coupling was strongest in both units, with Unit 2 showing greater sensitivity to pathological synchronization through higher coupling strength and larger waveform amplitudes. In contrast, during the interictal phase, AP-HFO coupling weakened in both units, with Unit 2 exhibiting a stronger suppressive effect (Unit 1: d = −0.548; Unit 2: d = −0.698). By the postictal phase, neither unit displayed significant changes in coupling, reflecting a recovery to baseline-like activity.

Detailed waveform analysis revealed distinctive morphological signatures across epileptic stages for both units (Figure 6f–i). Unit 1 maintained relatively stable waveform characteristics with modest changes in the coefficient of variation (CV) during interictal periods (1.14) compared to baseline (1.07) but showed marked instability during ictal periods (CV = 1.76). Conversely, Unit 2 exhibited more dynamic adaptations, with reduced CV during interictal periods (1.67 vs. baseline 1.99) followed by increases during ictal and postictal phases (1.95 and 1.92, respectively). These patterns were further supported by changes in waveform skewness and kurtosis, where Unit 2 displayed more pronounced shifts in distribution characteristics during ictal (kurtosis = 4.82) and postictal periods (kurtosis = 5.15) compared to Unit 1′s more modest modulations. The stability of these unit isolations throughout the recording was confirmed through consistent autocorrelograms and preserved refractory periods (Figure S9). These quantitative differences in waveform dynamics strengthen our observation that distinct neuronal subpopulations within CA1 contribute differentially to seizure dynamics.

While we focus on these two representative neuronal units from CA1 for detailed analysis, our probe successfully isolated neuronal units across multiple channels throughout the hippocampus. As shown in Figure S12, we recorded diverse unit waveforms from 54 active channels of the 128-channel probe, demonstrating the probe’s capability to record from different neuronal types across multiple hippocampal regions.

The inhibitory effects observed in CA1 during the interictal phase in Figure 6 may appear to contrast with the increased HFO and AP amplitudes observed in CA1 during the same phase in Figure 5. However, this apparent discrepancy reflects the inherent variability in seizure dynamics across experimental subjects and between individual neurons. The distinct waveform characteristics and coupling profiles of Unit 1 and Unit 2 exemplify how even neurons recorded from the same electrode can participate differently in epileptic networks. While Figure 5 and Figure 6 each present representative data from individual animals, the broader statistical analysis includes a sufficient sample size to account for such variability. This variability highlights the complexity of epileptic mechanisms, as different animals and individual neurons exhibit distinct interictal network dynamics, ranging from excitatory to inhibitory trends.

These findings underscore the limitations of relying solely on single metrics, such as amplitude or firing rates, to interpret seizure mechanisms. In contrast, the AP-HFO coupling analysis in Figure 6 provides a multidimensional perspective, capturing the diverse neuronal responses underlying epileptic activity. Furthermore, this analysis not only reflects the heterogeneity of neuronal activity but also reveals trends and severity of epileptic processes, offering a more comprehensive understanding.

Our previous research [28] successfully demonstrated gamma band synchronization changes and neuronal discharge temporal characteristics during epilepsy, establishing an important foundation for understanding epileptic network dynamics. The present study extends this foundation through the development of AP-HFO coupling analysis, enhancing our understanding of functional associations between neural signals. This multidimensional analytical perspective not only captures diverse neuronal responses but also reveals trends and severity of epileptic processes, thereby enhancing our ability to characterize the spatiotemporal features of epileptic activity.

## 4. Discussion

### 4.1. Advantages and Implications of Flexible Neural Probes

Our flexible probe represents a novel application of flexible electrode technology in epilepsy research, offering a powerful tool for investigating the mechanisms underlying seizure dynamics. Its high spatial resolution, achieved through sub-100 μm contact spacing, enables precise detection and localization of pathological HFOs [28,36], which are critical biomarkers of epileptic activity. In addition, the probe allows simultaneous detection of μV-scale HFOs and neuronal APs across interconnected hippocampal subregions, bridging network-level and cellular-level activity. This combination provides a comprehensive view of the multi-scale interactions involved in epilepsy, significantly enhancing the ability to study the hippocampus in greater detail. By facilitating investigations into how seizures originate and spread, the probe offers a more nuanced understanding of epileptic network dysfunction compared to traditional tools.

In the present study, we demonstrated the capability of the flexible probe in acute epilepsy experiments, showcasing its utility in investigating seizure dynamics. However, the flexible electrode is also well-suited for chronic neural recordings in epilepsy research. As shown in Figures S7 and S8, the probe achieved stable signal recordings over a period of four months, highlighting its long-term reliability. This stability further underscores its potential to advance epilepsy research and diagnostics by improving access to deep brain structures.

### 4.2. Spatiotemporal Dynamics of HFOs in the Hippocampus

The clustering of HFOs into distinct subtypes revealed important differences in their roles across baseline and ictal states. The subtypes with spectral energies concentrated in the 80–200 Hz range, which include Cluster Combined and Cluster 3, likely represent physiological synchronization mechanisms and may also involve early-stage pathological synchronization. In contrast, the subtype characterized by higher frequencies (>200 Hz) and increased amplitudes, defined as Cluster 4, exhibited a marked increase in quantity during the ictal phase. This subtype is strongly aligned with pathological synchronization mechanisms [37]. These findings support the hypothesis that pathological HFOs require higher levels of neuronal coupling and network excitability, consistent with previous studies [38].

Interestingly, during baseline conditions in the PTZ model, a small proportion of HFOs with characteristics of pathological synchronization (i.e., higher frequencies and increased amplitudes) were detected. This observation aligns with reports suggesting that chronic epilepsy models exhibit persistent pathological activity even in the absence of acute seizures [39]. These baseline pathological HFOs may reflect ongoing network instability, highlighting the potential of HFOs as biomarkers for detecting subclinical epileptic activity.

### 4.3. Stage- and Layer-Specific Contributions to Seizure Propagation

The dynamic characteristics of HFOs and APs across epileptic stages revealed distinct roles for the CA1 and CA3 hippocampal subfields. During the ictal phase, the gradual escalation of HFO firing rates in CA3 suggests its role as a seizure initiator, likely driven by its recurrent excitatory loops and intrinsic network excitability [40]. In contrast, CA1 displayed a delayed but more pronounced increase in HFO activity, reflecting its involvement in seizure propagation. These findings are consistent with the well-established feedforward excitation from CA3 to CA1 via Schaffer collaterals [41]. The heatmaps of HFO activity further demonstrated that CA3 exhibited sustained increases in the early ictal phase, while CA1 dynamically responded during seizure propagation.

Amplitude analyses of HFOs and APs further support this division of roles. The significant increase in both HFO and AP amplitudes during the ictal phase, particularly in CA3, underscores the heightened excitability and pathological synchronization of this region. By the postictal phase, the partial recovery of amplitudes suggests a gradual resolution of network hyperexcitability, although residual pathological activity in some animals may reflect differences in network recovery capacities. These observations emphasize the importance of spatial and temporal resolution in understanding seizure dynamics.

### 4.4. AP-HFO Coupling and Neuronal Heterogeneity

The coupling of APs with HFOs varied significantly across epileptic stages and hippocampal layers, reflecting distinct mechanisms of neuronal synchronization. During the ictal phase, AP-HFO coupling was strongest in CA3, consistent with its role as the primary driver of seizure activity [42]. This coupling likely reflects the recruitment of neuronal populations into synchronized activity, facilitating the propagation of seizures. In the interictal phase, weaker AP-HFO coupling was observed, indicating a partial decoupling of neuronal activity from pathological synchronization. Notably, in CA1, a potential inhibitory effect of HFOs on AP firing was detected, suggesting that local inhibitory circuits may play a role in modulating excitability during the interictal phase.

At the single-neuron level, significant heterogeneity in AP-HFO coupling strength was observed. Unit 2, characterized by larger waveform amplitudes and stronger responses to HFOs, exhibited greater sensitivity to pathological synchronization compared to Unit 1. This heterogeneity highlights the diversity of neuronal responses to HFO events, even within the same recording location. These findings suggest that only a subset of neurons actively participates in pathological synchronization, while others may contribute to stabilizing or inhibiting network activity [43,44]. This diversity underscores the complexity of local network dynamics during seizures and highlights the need for multi-scale analyses to fully understand epileptic mechanisms.

## 5. Conclusions

This study highlights the spatiotemporal dynamics of HFOs and APs across epileptic stages, revealing distinct roles for hippocampal subfields in seizure initiation and propagation. Leveraging a custom-designed flexible deep-brain probe, we achieved simultaneous high-resolution recordings of HFOs and APs, enabling unprecedented insights into hippocampal network dysfunction during seizures. The identification of pathological HFOs as biomarkers for ictal activity and the observed neuronal heterogeneity in AP-HFO coupling provide new perspectives on the complexity of epileptic network dynamics. These findings have important implications for the diagnosis and treatment of epilepsy, emphasizing the need for spatially and temporally targeted interventions to modulate seizure activity.

## Data Availability

The original contributions presented in this study are included in the article. Further inquiries can be directed to the corresponding author.

## Supplementary Materials

The following supporting information can be downloaded at: https://www.mdpi.com/article/10.3390/mi16060661/s1, Figure S1: Microscopic images of the flexible electrode before and after electrochemical coating; Figure S2: Assembled 128-channel flexible electrode; Figure S3: Fabrication process of the dual-side flexible electrode; Figure S4: HFO firing rates in two additional mice after PTZ injection; Figure S5: Heatmaps of HFO firing rates in KA-induced epileptic mice; Figure S6: Immunohistochemical images of various implantable devices after 8 weeks post implantation; Figure S7: Stable long-term recording of neural action potentials from different hippocampal regions over four months; Figure S8: Long-term recording and analysis of neural signals from a single channel over four months; Figure S9: ACGs and ISI distributions for Units 1 and 2 as shown in Figure 6f–i; Figure S10: Assembly configuration showing the flexible electrode bonded to tungsten wire support structures, demonstrating the dual-shank design that provides sufficient rigidity for precise implantation; Figure S11: Impedance measurements of the dual-sided probe’s 128 channels before and after PEDOT:PSS electroplating; Figure S12: Waveforms of isolated units recorded from 54 active channels of the 128-channel probe; Figure S13: Correspondence between the electrode array implantation schematic and neuronal firing rate heatmap; Table S1: Comparison of SEEG, ECoG, and the Flexible probe in this study.
