# Supplementary material for: Flexible Deep-Brain Probe for High-Fidelity Multi-Scale Recording of Epileptic Network Dynamics
Source: Micromachines (Basel). 2025 May 30;16(6):661. doi: 10.3390/mi16060661 (PMC12195220; doi:10.3390/mi16060661)
Supplement: Supplementary file 1 [file micromachines-16-00661-s001.zip › micromachines-3630753-supplementary.pdf]

Article

Flexible Deep-Brain Probe for High-Fidelity Multi-Scale Recording of Epileptic Network Dynamics

Dujuan Zou [1,2,†], Lirui Yang [1,2,†], Guopei Zhou [1,3], Yan Zhang [1,2], Zhenyu Liang [1,2], Ziyi Zhu [1], Yanyan Nie [4], Huiran Yang [1], Zhitao Zhou [1,2], Liuyang Sun [2,5] and Xiaoling Wei [1,2,*]

1 State Key Laboratory of Transducer Technology, Shanghai Institute of Microsystem and Information Technology, Chinese Academy of Sciences, Shanghai 200050, China; dujuan@mail.sim.ac.cn (D.Z.); yanglirui24@mails.ucas.ac.cn (L.Y.); boblezhou@gmail.com (G.Z.); zhangyan1@mail.sim.ac.cn (Y.Z.); lzy0221@mail.sim.ac.cn (Z.L.); zhuzy22@mail.sim.ac.cn (Z.Z.); hryang@mail.sim.ac.cn (H.Y.); ztzhou@mail.sim.ac.cn (Z.Z.)
2 School of Graduate Study, University of Chinese Academy of Sciences, Beijing 100049, China; liuyang.sun@mail.sim.ac.cn
3 Wuhan Research Institute of Posts and Telecommunications, Wuhan 430074, China
4 Shanghai Laboratory Animal Research Center, Shanghai 201203, China; nieyanyan@slarc.org.cn
5 2020 X-Lab, Shanghai Institute of Microsystem and Information Technology, Chinese Academy of Sciences, Shanghai 200050, China
* Correspondence: xlwei-jerry@mail.sim.ac.cn; Tel.: +86-21-62511070
† These authors contributed equally to this work.

Table S1. Comparison of SEEG, ECoG, and the Flexible probe in This Study.

Characteristics	SEEG	ECoG	This work
Number of Channels	Low (4–18) [1–4]	Moderate (6–64) [5,6]	High (128)
Tissue Damage	Moderate (footprint: 0.50 or 0.58 mm^2) [1–4]	Moderate (cranial window:>240 mm^2)	Low (footprint: 113.5 μm^2)
Recording Stability	Low (8-26 days) [7–9]	Low (10-29 days) [10–12]	High (>4 months)

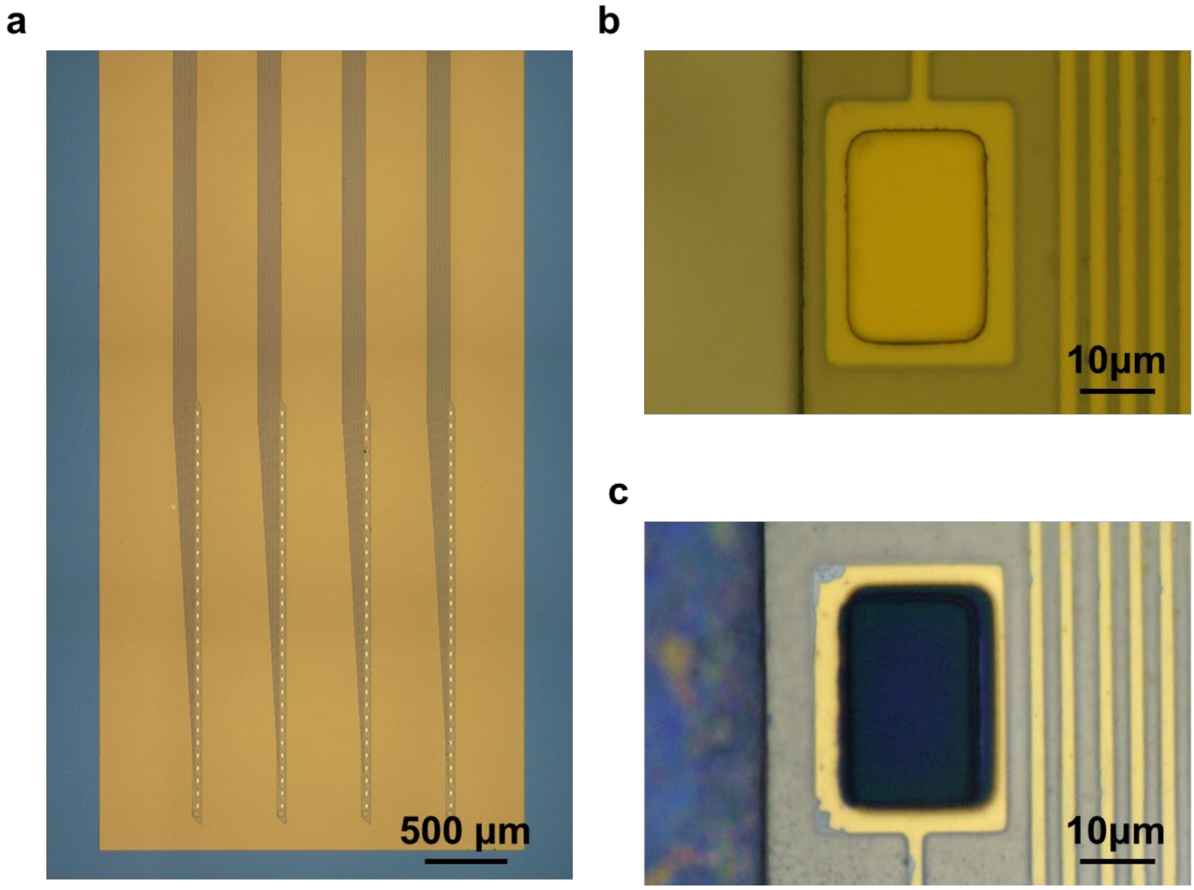

Figure S1. Microscopic images of the flexible electrode before and after electrochemical coating. (**a**) Micrograph of the flexible electrode tip showing the four shanks and the 128 recording sites. This image provides a detailed view of the electrode's configuration, with the four shanks and recording sites visible at the tip. (**b**) Photograph of the electrode recording sites before electrochemical coating, showing the gold (Au) electrode sites. This image displays the electrode's appearance prior to the application of the electrochemical coating. (**c**) Photograph of the electrode after electrochemical coating with PEDOT:PSS. This image shows the electrode after the PEDOT:PSS coating, demonstrating the modified surface for improved electrode performance.

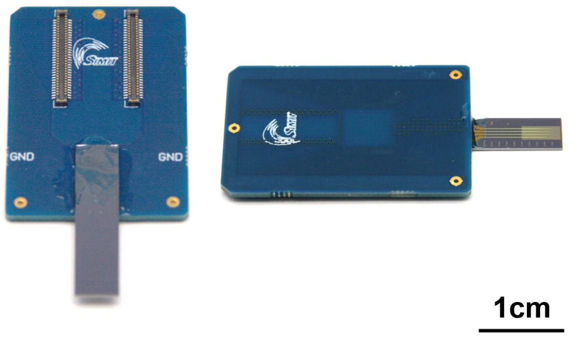

Figure S2. Assembled 128-channel flexible electrode. The right side shows the front of the electrode, while the left side displays the back of the electrode. At this stage, the sacrificial layer has not been removed.

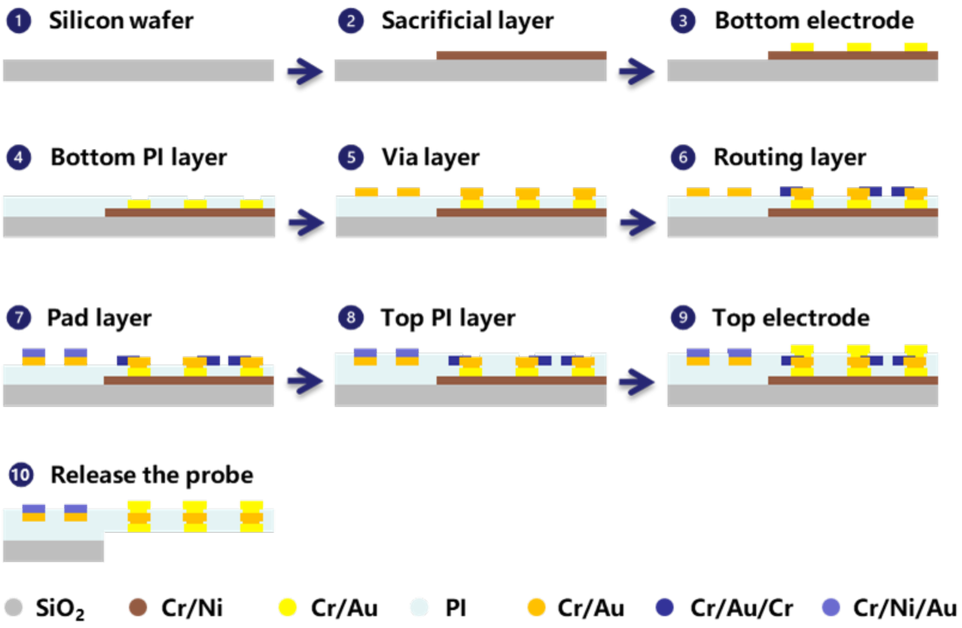

Figure S3. Fabrication process of the dual-side flexible electrode.

The bending stiffness equation is given as:

$$K = E_s \frac{wh^3}{12} \quad (1)$$

Where K is the bending stiffness, E_s is the Young's modulus, w is the probe width, and h is the thickness. Substituting E_s = 2.5 GPa, w = 113.5 μm, and h = 1 μm into the equation, the bending stiffness is calculated as $K = 2.36 \times 10^{-14}$ N·m², indicating extremely high flexibility.

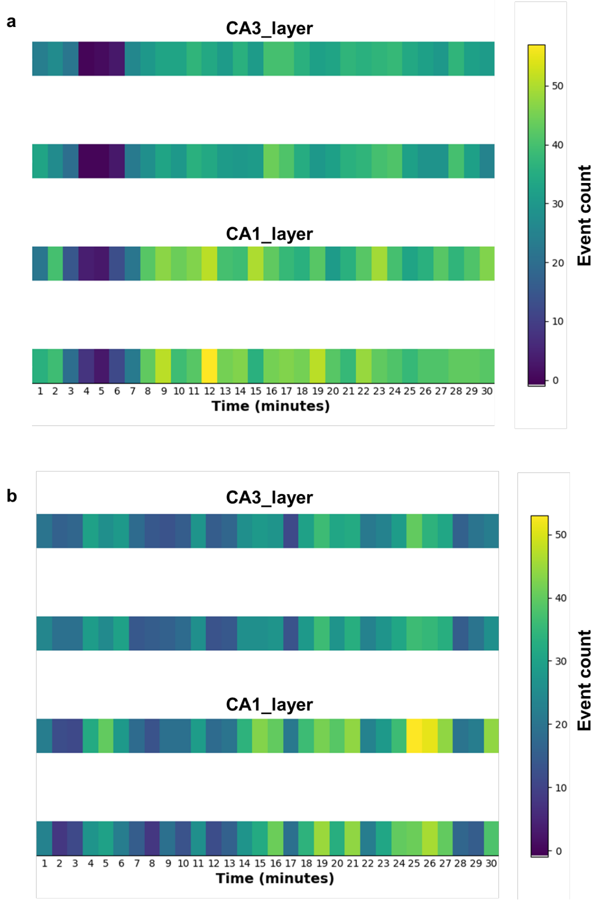

Figure S4. HFO firing rates in two additional mice after PTZ injection. (a) First mouse's HFO activity in CA1 and CA3 regions. (b) Second mouse's HFO activity showing similar patterns, although seizure onset was delayed in this mouse. Despite individual variations, the overall trend remained consistent across all animals. Both datasets show that CA1 exhibited higher seizure intensity compared to CA3, indicating increased sensitivity to seizures. The firing rates increased and then declined as seizures progressed, reflecting typical dynamic changes during the seizure process. These results confirm the consistency of observations across animals.

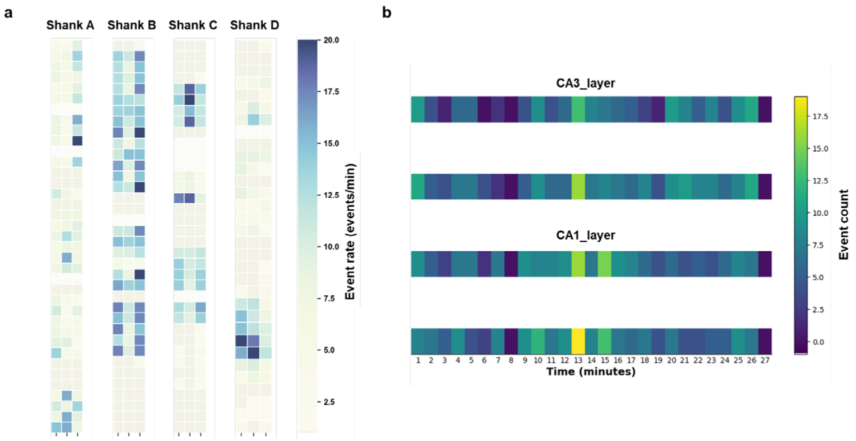

Figure S5. Heatmaps of HFO firing rates in KA-induced epileptic mice. (a) Heatmap showing the HFO firing rates detected across 128 channels in kainic acid (KA)-induced epileptic mice (1 μL of 1 μg/μL). (b) Representative heatmaps of HFO firing rates from two channels in CA1 and two channels in CA3, illustrating the entire duration of the seizure from onset to termination.

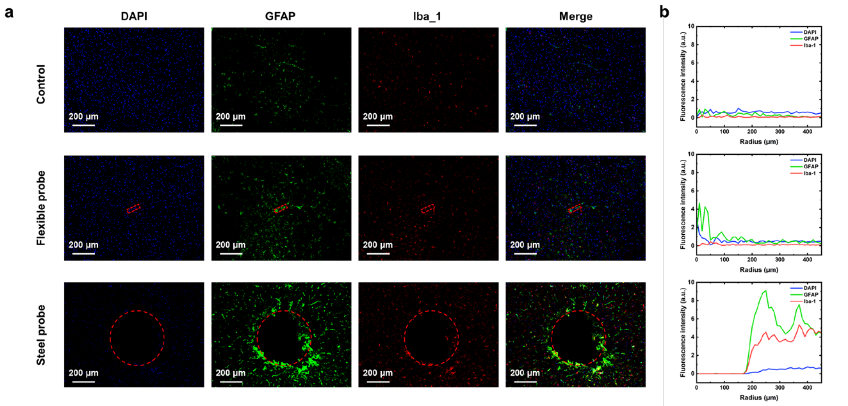

Figure S6. Immunohistochemical images of various implantable devices after 8 weeks post implantation. (a) Confocal fluorescence images of 20 μm-thick tissue slices, allowing the visualization of astrocytes (GFAP, green), microglia (Iba-1, red), and cell nuclei (DAPI, blue). (b) The fluorescence intensities of DAPI, GFAP, and Iba-1 as a function of the radial distance from the center of implantation, measured by averaging fluorescence intensities along concentric circles centered at the implantation site for stainless steel wire electrodes and flexible neural electrodes.

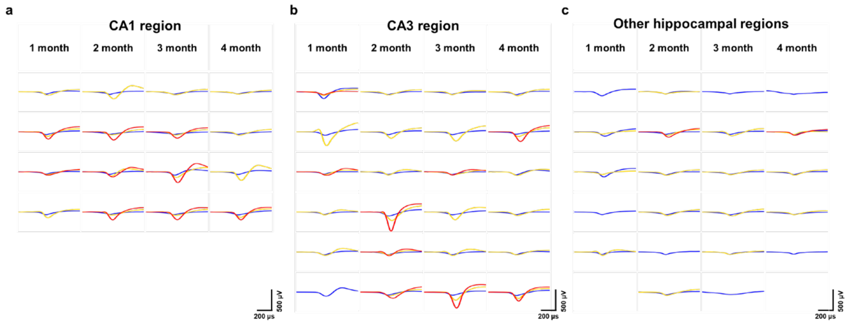

Figure S7. Stable long-term recording of neural action potentials from different hippocampal regions over four months. (a) displays neural signals recorded from four channels in the CA1 region. (b) shows signals recorded from six channels in the CA3 region. (c) presents signals recorded from hippocampal regions outside of CA1 and CA3. This figure illustrates neural signals recorded from one mouse over a four-month period, showcasing the capability of the electrode to consistently capture and isolate neuronal action potentials across different regions of the hippocampus.

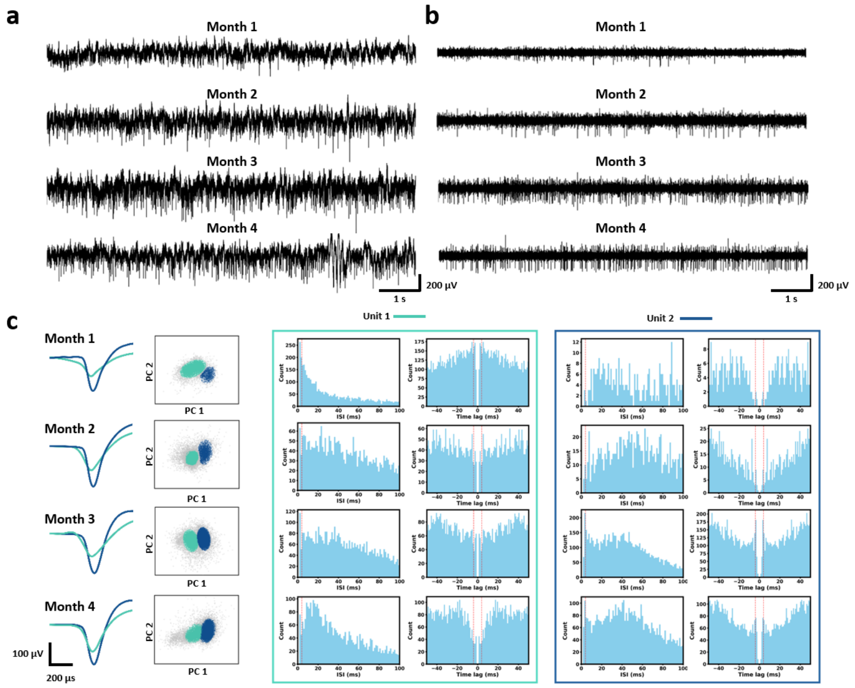

Figure S8. Long-term recording and analysis of neural signals from a single channel over four months. (a): Raw neural signals (10 s) recorded from the channel before filtering. (b): Filtered neural signals (10 s) from the same channel after applying a 250 Hz high-pass filter. (c): Analysis of unit separation and activity (based on 8 minutes of data). From left to right: The first column displays the overlaid waveforms of Unit 1 and Unit 2, where the dark blue line represents the average waveform of Unit 2, and the green line represents the average waveform of Unit 1. The second column shows the PCA clustering results, where dark blue dots represent Unit 2 data points, and green dots represent Unit 1 data points. The third and fourth columns present the ISI (inter-spike interval) and ACG (autocorrelogram) for Unit 1, respectively. The fifth and sixth columns show the ISI and ACG for Unit 2, respectively.

In the ISI plots, the red dashed line indicates the 2 ms refractory period, which is a critical metric for assessing the quality of unit isolation. Generally, if the proportion of events within 2 ms is below 2%, the unit isolation quality is considered acceptable. Our results demonstrate that the waveforms recorded from this channel meet the 2% threshold within the 2 ms refractory period, indicating high-quality unit isolation.

The ACGplots provide insight into the temporal structure of spike trains, revealing characteristics such as periodicity, burst firing, or randomness in spike activity. Over the four months of recording, Unit 1 displayed a transition in its ACG pattern, evolving from a high-center, low-sides distribution in the first month (indicative of burst firing) to a low-center, high-sides distribution from the second to fourth months (reflecting periodic firing and increased network synchronization). In contrast, Unit 2 consistently exhibited a low-center, high-

sides pattern throughout the four-month period, suggesting its persistent involvement in synchronized network activity. These ACG patterns reflect dynamic changes in neural activity associated with epileptic network remodeling over time.

This figure highlights the robustness of the electrode in maintaining stable, high-quality recordings over four months, enabling reliable unit isolation and providing valuable insights into long-term neural network dynamics and pathological changes.

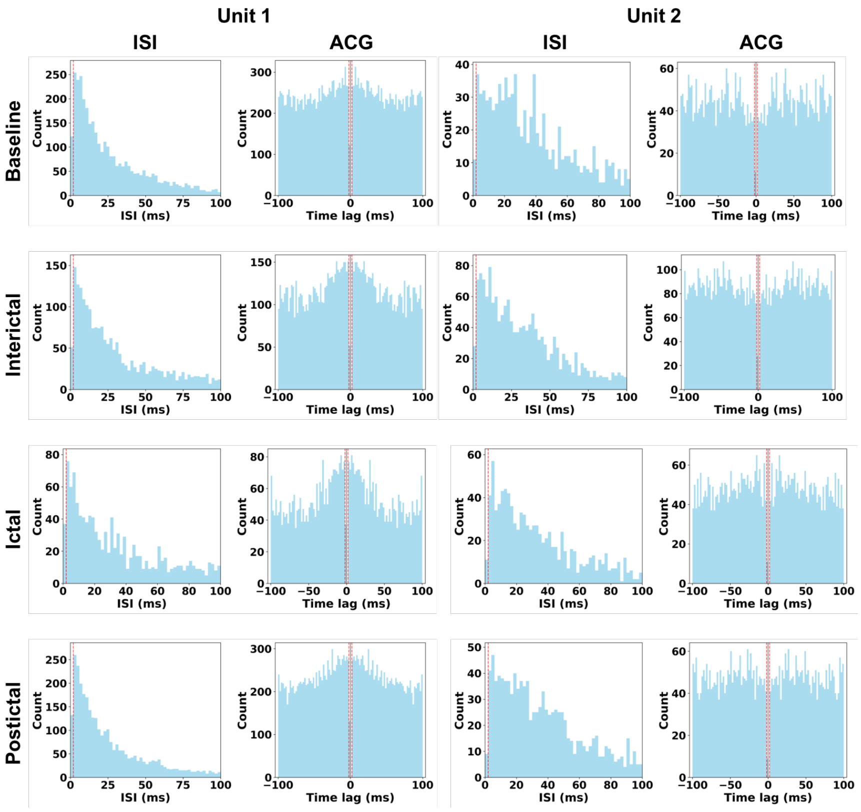

Figure S9. ACGs and ISIdistributions for Units 1 and 2 as shown in Figure 6f-i. The consistent ACG patterns and preserved refractory periods (<2ms, red dashed lines) confirm that signals originate from the same neurons throughout the recording, despite amplitude changes during seizures. The stable unit isolation validates that amplitude fluctuations in Unit 2 represent genuine neurophysiological responses rather than signal contamination.

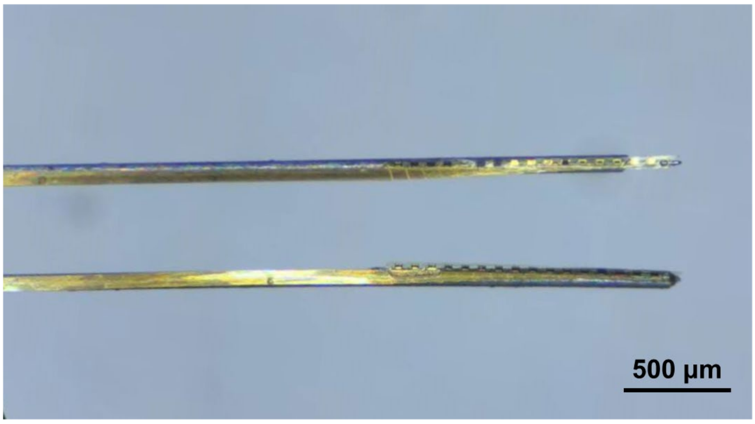

Figure S10. Assembly configuration showing the flexible electrode bonded to tungsten wire support structures, demonstrating the dual-shank design that provides sufficient rigidity for precise implantation.

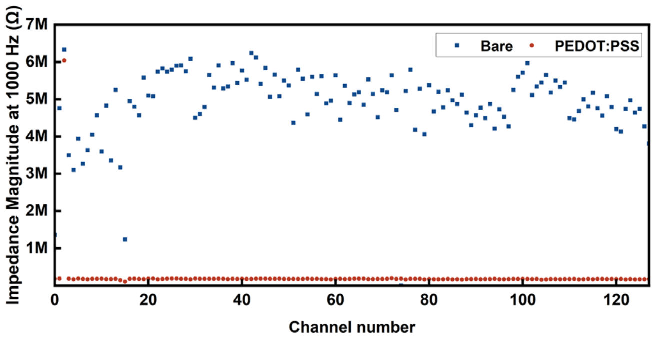

Figure S11. Impedance measurements of the dual-sided probe's 128 channels before and after PEDOT:PSS electroplating. The data demonstrates a high functional yield of 99.2% (127/128 channels), with successful impedance reduction across channels after electroplating. This high yield validates the effectiveness of our sequential multi-layer microfabrication approach for dual-sided probe fabrication.

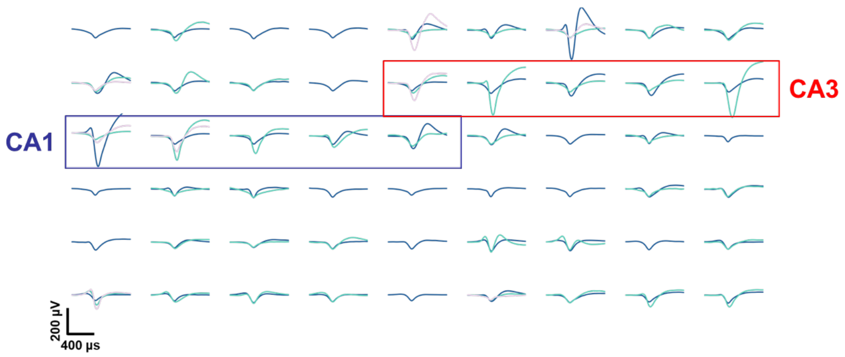

Figure S12. Waveforms of isolated units recorded from 54 active channels of the 128-channel probe. The diverse waveforms demonstrate the probe's capability to record from different neuronal types across multiple hippocampal regions.

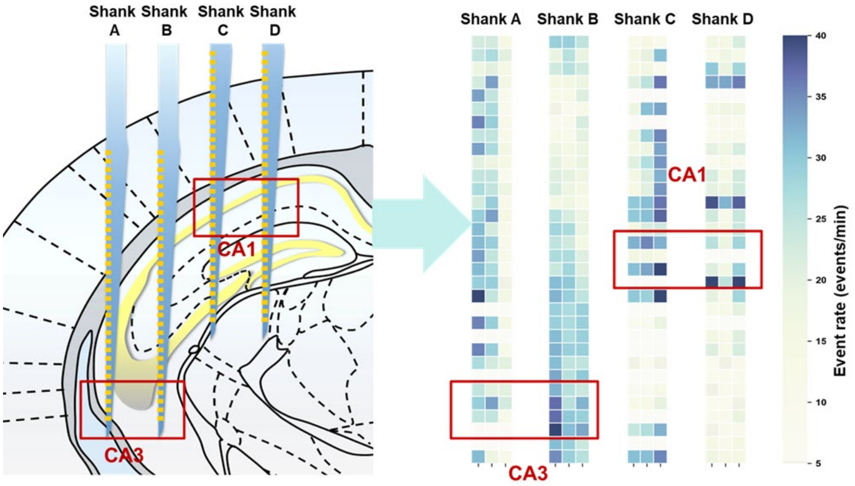

Figure S13. Correspondence between the electrode array implantation schematic and neuronal firing rate heatmap. The implantation schematic indicates the CA1 and CA3 regional areas, which are correspondingly marked on the firing rate heatmap, revealing distinct neuronal activity patterns across hippocampal subregions during seizure events. This spatial mapping provides intuitive visualization of how neural activity correlates with hippocampal anatomical structures.

Reference:

1. Curot, J. *et al.* Local neuronal excitation and global inhibition during epileptic fast ripples in humans. *Brain* **146**, 561–575 (2023).
2. Köksal-Ersöz, E. *et al.* Whole-brain simulation of interictal epileptic discharges for patient-specific interpretation of interictal SEEG data. *Neurophysiologie Clinique* **54**, 103005 (2024).
3. de Andrade Morange, D. *et al.* Hippocampal intracerebral evoked potentials as a marker of its functionality in drug-resistant epilepsy. *Neurophysiologie Clinique* **52**, 323–332 (2022).
4. Kassiri, J. *et al.* Safety and Efficacy of Stereoelectroencephalography in Pediatric Epilepsy Surgery. *Journal of Pediatric Epilepsy* **11**, 75–79 (2022).
5. Boran, E. *et al.* High-density ECoG improves the detection of high frequency oscillations that predict seizure outcome. *Clinical Neurophysiology* **130**, 1882–1888 (2019).
6. Won, S.-Y. *et al.* DIagnostic Subdural EEG electrodes And Subdural hEmatoma (DISEASE): a study protocol for a prospective nonrandomized controlled trial. *Neurological Research and Practice* **2**, 50 (2020).
7. Bottan, J. S. *et al.* Lack of spontaneous typical seizures during intracranial monitoring with stereo-electroencephalography. *Epileptic Disorders* **25**, 833–844 (2023).
8. Pothof, F. *et al.* Chronic neural probe for simultaneous recording of single-unit, multi-unit, and local field potential activity from multiple brain sites. *J. Neural Eng.* **13**, 046006 (2016).
9. Ouchida, S., Nikpour, A., Wilson, D. N. & Fairbrother, G. Case Report: Ictal hypersalivation: a stereoelectroencephalography exploration. *Front. Surg.* **12**, (2025).
10. Freund, B. E. *et al.* Early Postoperative Seizures Following Awake Craniotomy and Functional Brain Mapping for Lesionectomy. *World Neurosurgery* **181**, e732–e742 (2024).
11. Inoue, T. *et al.* Distinct dual cortico-cortical networks successfully identified between supplemental and primary motor areas during intracranial EEG for drug-resistant frontal lobe epilepsy. *Epilepsy & Behavior Reports* **15**, 100429 (2021).
12. Chiba, R. *et al.* Usefulness of Intraoperative Electrocorticography for the Localization of Epileptogenic Zones. *Neurologia medico-chirurgica* **63**, 65–72 (2023).
